# Parent Learning Groups in Alternative Provision: A Mixed-Methods Study of Psychoeducation, Mentalization, and Peer Support for Parents of Children with Neurodevelopmental and Conduct Difficulties

**DOI:** 10.3390/children13030431

**Published:** 2026-03-21

**Authors:** Gali Chelouche-Dwek, Peter Fonagy

**Affiliations:** 1 Anna Freud Centre, London N1 9JH, UK; 2 Research Department of Clinical, Educational and Health Psychology, University College London, London WC1E 6BT, UK; p.fonagy@ucl.ac.uk

**Keywords:** mentalization-based intervention, parental reflective functioning, parenting self-efficacy, alternative provision, parent learning group, psychoeducation, neurodevelopmental difficulties, school-based intervention, mixed methods, epistemic trust

## Abstract

**Highlights:**

**What are the main findings?**

• A school-embedded parent learning group integrating psychoeducation, mentalization-based practice, and peer support was associated with large improvements in parental reflective functioning and parenting self-efficacy.
• Parents showed reduced pre-mentalizing and increased curiosity about their children’s mental states, alongside greater confidence in managing challenging behaviour.
• Qualitative analysis identified six interconnected themes, including relational safety, enhanced mentalizing capacity, improved affect regulation, and transformed parent–child interactions, elucidating the mechanisms of change.

**What are the implications of the main findings?**

• School-based mentalization-informed parent groups are a feasible and accessible intervention model for families of children with neurodevelopmental and conduct difficulties who are not engaged with clinical services.
• Embedding parent support within schools may strengthen parenting capacity while transforming adversarial parent–school relationships into collaborative partnerships.
• Improvements in parental mentalizing may contribute to children’s emotional regulation and engagement by enhancing relational safety and containment.

**Abstract:**

Background: Parents of school-age children with neurodevelopmental and conduct difficulties face elevated stress, reduced self-efficacy and relational strain, yet evidence for scalable, school-embedded support remains limited. Drawing on mentalization theory—which emphasises parents’ capacity to understand behaviour in terms of underlying mental states—this mixed-methods study evaluated a weekly parent learning group integrating psychoeducation, mentalization-based practice and peer support, delivered within an alternative provision school. Methods: A group of twelve parents who attended at least six sessions completed retrospective pretest–posttest questionnaires assessing parental reflective functioning (PRFQ) and parenting self-efficacy (PSOC). Semi-structured interviews explored parents’ subjective experiences and perceived changes in parent–child interactions and parent–school relationships. Quantitative outcomes were analysed using paired *t*-tests and effect sizes; qualitative data underwent reflexive thematic analysis. Results: Quantitative analyses revealed statistically significant improvements in parental reflective functioning and self-efficacy. Pre-mentalizing scores decreased substantially (*d* = 1.34), indicating reductions in non-mentalizing, while interest and curiosity about children’s mental states increased markedly (*d* = 1.83). Parenting self-efficacy improved significantly (*d* = 1.61). Although a reduction in excessive certainty about mental states approached significance (*d* = 0.63, *p* = 0.053), trends suggested greater epistemic balance. Qualitative analysis identified six themes elucidating mechanisms of change, including enhanced mentalizing capacity, reduced parental stress, transformed parent–child interactions and facilitation style as a critical active ingredient. Integration of findings suggests that psychoeducational content provided conceptual grounding for understanding behaviour, facilitator modelling scaffolded reflective practice, and relational safety within the group enabled authentic engagement with challenging experiences. Conclusions: These preliminary findings indicate that a school-based parent learning group combining psychoeducation, mentalization-based practice and peer support is feasible and associated with meaningful improvements in parental reflective functioning and self-efficacy. Parent narratives of transformed relational practices and shifts from reactive to reflective engagement echo broader literature demonstrating that group-delivered mentalization-oriented programmes can enhance reflective capacities and caregiving quality in diverse family contexts. The school setting may extend the reach of such interventions to families not engaged with clinical services and support collaborative parent–school partnerships. Future research should employ larger, controlled designs, incorporate observational and child outcome measures, and explore scalability across educational contexts.

## 1. Introduction

### 1.1. Rationale for School-Based Mentalization Support for Parents

Schools, and alternative provision settings in particular, represent a critical yet underutilised context for delivering mentalization-informed parent support. Most research on mentalization-based parenting interventions has been conducted within clinical Child and Adolescent Mental Health Services (CAMHS) or with parents of infants and young children [1,2], despite compelling evidence that school-age children with neurodevelopmental and conduct difficulties and their families face distinct challenges that clinical services alone cannot adequately address. Parents of children placed in alternative provision frequently experience interactions with schools as adversarial and feel blamed or stigmatised for their child’s behaviour [3], yet these same parents are often disengaged from clinical pathways. Embedding parent support within the school positions the educational institution as a collaborative partner rather than an additional source of stress, potentially transforming relational dynamics that have historically undermined family engagement [4].

The present study was designed to address this gap by evaluating a parent learning group that integrates psychoeducation, mentalization-based practice and peer support within an alternative provision school—a combination of intervention components and delivery context that, to our knowledge, has not been previously investigated.

Critically, while mentalization-based approaches have generated considerable theoretical interest and emerging empirical support, the evidence base for group-delivered mentalization interventions remains at an early stage. Systematic reviews have noted that studies are methodologically heterogeneous, sample sizes are often small, controlled designs are rare, and long-term follow-up data are largely absent [1,2,5]. The present study, therefore, positions itself not as extending well-established intervention effects but as contributing to an evolving and still limited evidence base—one that requires rigorous, context-sensitive research to determine whether and how mentalization-informed parent support can be effectively delivered outside clinical settings.

### 1.2. The Prevalence and Impact of Emotional and Behavioural Difficulties in Children

Emotional and behavioural problems in children are among the most common causes of functional disability in childhood and represent a significant public health concern across diverse cultural and socio-environmental contexts [6,7]. One in three mental disorders develops before the age of 14, with middle childhood (5–12 years) representing a crucial developmental period during which symptoms of emotional distress frequently emerge [7].

In England, approximately one in six children aged 5 to 16 has been identified as having a probable mental disorder, yet access to effective mental health services remains severely limited, leading to a substantial treatment gap [7,8]. The majority of children presenting to Child and Adolescent Mental Health Services (CAMHS) display mixed emotional and behavioural difficulties characterised by high rates of transdiagnostic co-morbidity, yet most empirically tested treatments have been developed for single disorders [8]. Children with such complex presentations frequently challenge mainstream educational provision and may be placed in alternative provision settings, where their needs intersect with those of their families in ways that demand comprehensive, multi-component interventions.

### 1.3. The Role of Parents and Family Systems

Research consistently demonstrates that parenting plays a critical role in helping children become well-adjusted, with the quality of parent–child interactions during early years being especially significant for socio-emotional development [6,9]. Family-level factors such as parental stress, mental health difficulties, social isolation, and adverse experiences can significantly compromise parenting capacity and exacerbate children’s emotional and behavioural difficulties [10,11]. For example, research conducted in Finland linked concerns about child and adolescent well-being and mental health to increased rates of parental divorce, poverty, substance abuse, and mental health problems, all of which elevate the risk for child neglect and disrupted parent–child relationships [11].

Parents of children with neurodevelopmental conditions and emotional difficulties frequently experience elevated stress, reduced self-efficacy, and feelings of helplessness, particularly when parenting strategies fail to produce desired outcomes [12,13]. These cumulative adversities underscore the need for interventions that address not only children’s difficulties but also the emotional and relational needs of their parents.

### 1.4. Group-Based Parent Training and Psychoeducational Interventions

Cochrane systematic reviews have established robust evidence that group-based parent training programmes are effective in improving the emotional and behavioural adjustment of young children and may serve a role in the primary prevention of emotional and behavioural problems [6,9]. Barlow et al. [6] reviewed randomised controlled trials examining group-based parenting programmes for children up to approximately four years of age, demonstrating significant improvements in child emotional and behavioural outcomes. An earlier review focusing specifically on children from birth to three years similarly found that parenting programmes delivered in group formats positively impact emotional and behavioural adjustment in infants and toddlers [9].

Psychoeducational approaches, involving the systematic provision of information about a child’s condition alongside strategies for management and coping, have been increasingly applied to diverse populations of parents. Gearing [14] reviewed the evidence for family psychoeducational interventions, finding that such approaches consistently impact families positively and, in the context of psychotic disorders, reduce relapse rates by improving social and occupational functioning. Wagner [15] similarly identified parent training, support, and psychoeducational groups as evidence-based practices in children’s mental health, noting that despite the significant expansion of the evidence base, implementation of these programmes has lagged behind, particularly in rural and underserved communities.

Empirical studies have demonstrated the feasibility and effectiveness of psychoeducational parenting groups across varied settings. Berge et al. [12] conducted a pilot study testing a 12-week psychoeducational parenting group within a primary care clinic serving an underserved population. Participants reported statistically significant improvements in family functioning, reductions in child misbehaviour, and enhanced couple functioning, suggesting that integrated, group-based psychoeducational approaches can effectively address child behavioural problems in non-specialist settings.

### 1.5. The Helping Families Programme and Psychoeducational Interventions

The Helping Families Programme [10] represents a related approach specifically designed for parents affected by clinically significant personality difficulties. This 16-week intervention works collaboratively with parents to explore how their emotional and relational difficulties impact parenting and child functioning, illustrating the growing recognition that effective psychoeducation must address not only knowledge acquisition but also the interpersonal and emotional dimensions of parenting. More recently, Guerra et al. [13] conducted a systematic review of parenting interventions for children with specific learning disorders, finding that programmes combining emotional support with educational approaches improved parents’ wellbeing and coping strategies, which in turn were linked to better academic, behavioural and social outcomes in children. Together, these studies demonstrate that psychoeducational parent groups can be effective across diverse clinical populations and service contexts when they address both the informational and emotional needs of parents.

### 1.6. Mentalization-Based Approaches for Parents and Families

Mentalization—the capacity to understand behaviour in terms of underlying mental states such as thoughts, feelings, desires and intentions [16]—has emerged as a central construct in understanding parent–child relationships and the development of children’s emotional regulation capacities. Mentalization-based treatment (MBT), originally developed for adults with personality disorders, has been increasingly adapted for work with children, adolescents and families [1,8,17]. Byrne et al. [1] conducted a systematic review identifying 34 MBT studies across these populations, including interventions focused on parent–child dyads, parenting groups, school environments and adolescent populations. The review found tentative support for MBT approaches in increasing mentalizing and reflective functioning, while noting the need for further controlled and methodologically rigorous research. Midgley et al. [2] conducted a narrative systematic review of mentalization-based interventions specifically for children aged 6–12 and their carers, identifying 29 unique interventions. Critically, they noted that the evidence base for this age group remains substantially underdeveloped compared with evidence for infants or adults, with only a third of identified publications reporting outcome data—and among those that did, methodological heterogeneity was considerable, with varying outcome measures, intervention formats and comparison conditions limiting the synthesis of findings. More recently, Kalland et al. [5] observed in an editorial on new frontiers in parental mentalization that while the field has expanded rapidly, many studies remain small-scale, uncontrolled and lacking in long-term follow-up, underscoring the need for methodologically rigorous research to determine which elements of mentalization-informed interventions produce sustainable change and under what conditions.

Several mentalization-based group programmes have demonstrated promising results for parents. The Lighthouse Parenting Program [18] evaluated a 12-week group intervention designed to promote mentalization in parents facing difficulties in the parent–child relationship. Results from 101 participants revealed significant improvements in mentalizing, parental adjustment and family functioning, alongside reductions in coercive parenting practices. The programme emphasised curiosity about children’s minds and willingness to reflect on one’s own feelings, thoughts and behaviours—core principles of mentalization-based work. Similarly, the Families First intervention [11] was developed as a mentalization-based group model for first-time parents in Finland, aiming to support well-functioning models of parenting and prevent the intergenerational transmission of negative parenting patterns. Both programmes illustrate how group-based mentalization approaches can enhance parental reflective capacity while simultaneously providing peer support that reduces isolation and normalises parenting challenges.

Beyond individual-level mechanisms, the group format itself represents a theoretically meaningful therapeutic context. Mentalization-Based Group Therapy (MBT-G), as articulated by Karterud [19], describes how group settings can cultivate a mentalizing group culture—an interpersonal environment in which shared curiosity about mental states becomes a group norm, epistemic openness is reinforced through inter-member interaction, and the relational fabric of the group itself becomes a medium through which mentalizing is activated and sustained. In this framework, the group is not merely a cost-efficient delivery vehicle for content that could equivalently be provided individually; rather, the group setting generates distinctive therapeutic processes, including inter-member modelling of reflective stances, the normative influence of shared exploration, and the corrective emotional experience of being understood by peers who share one’s difficulties. These processes resonate with classical and enduring formulations of group therapeutic factors by Yalom and Leszcz [20]—particularly universality (the recognition that one’s struggles are widely shared), group cohesion (a sense of belonging and mutual acceptance), altruism (the experience of meaningful contribution to others’ wellbeing), and instillation of hope—each of which may independently and additively support reflective functioning and parenting self-efficacy in group-based parent interventions [21]. The present study offers an opportunity to examine whether these group-level processes, alongside specific psychoeducational and mentalization content, may contribute to observed outcomes.

The Emotion Regulation in Children (ERiC) trial [8] represents the first large-scale randomised controlled trial evaluating MBT for school-age children with mixed emotional and behavioural difficulties. The protocol describes a transdiagnostic approach in which MBT is compared with treatment as usual within CAMHS settings, with sessions offered fortnightly and flexibly incorporating different family members. The ERiC trial recognises that mentalizing may help with emotional regulation across a range of clinical presentations, addressing the research–practice gap for children presenting with co-morbid difficulties. This trial, alongside the evidence from group-based programmes, suggests that mentalization-based approaches hold considerable promise for families of children with complex emotional and behavioural needs, while simultaneously highlighting that the field has yet to produce the volume of controlled, replicable evidence required to consider these interventions well-established.

### 1.7. School-Based Interventions and Mentalization in Educational Contexts

Schools represent a critical context for the delivery of mental health interventions, offering access to large populations of children within their natural environment [22]. Brown et al. [7] synthesised evidence from 52 intervention studies and 53 systematic reviews, identifying several promising approaches for children aged 5–12 experiencing emotional and behavioural difficulties, including cognitive–behavioural techniques, social-emotional learning programmes and parent-focused components. Hayes et al. [23] conducted a meta-analysis of 71 studies involving over 63,000 participants and found that universal school-based interventions produced small but significant improvements in anxiety and depression outcomes, with cognitive–behavioural approaches demonstrating greater effectiveness than mindfulness-based programmes for anxiety specifically. These reviews establish that school-based approaches can produce meaningful, population-level benefits for children’s emotional wellbeing, though the evidence base for targeted, indicated interventions for children with complex difficulties remains limited.

Mentalization-based interventions (MBIs) have been increasingly applied within school settings to support socio-emotional development and mental health. Chelouche-Dwek and Fonagy [24] conducted a systematic review of MBIs implemented in educational contexts for students aged 6–18, identifying 21 studies comprising over 7500 participants. The reviewed interventions targeted various aspects of mentalizing, including emotion-understanding, empathy, perspective-taking and Theory of Mind. Significant improvements were found in social-cognitive abilities, classroom behaviour, well-being, and peer relationships. Midgley et al. [2] similarly identified several MBT interventions that work with whole systems, including schools, noting their potential for reaching children who might not otherwise access clinical services. These findings, combined with the ERiC trial’s [8] recognition of the need for transdiagnostic approaches, suggest that school settings represent a viable and promising context for mentalization-based work with children and their families.

### 1.8. Parent–School Communication and Partnership

Effective collaboration between parents and schools is essential for supporting children with emotional and behavioural difficulties, yet significant barriers to productive communication persist, particularly for families of children with complex needs. Buchanan and Clark [3] conducted qualitative interviews with parents and teachers of students with emotional and behavioural disorders, finding that parents frequently feel blamed or stigmatised for their children’s behaviour and often experience interactions with schools as adversarial. Their study revealed that parents’ perceptions of communication quality varied by school type, while both parents and teachers identified barriers related to school culture, prior negative experiences, technology, institutional constraints and limited interest in engaging.

Walker et al. [4] similarly emphasised that special education requires schools, communities and service providers to come together as partners with families, positioning families as the most enduring, expert supporters of their children. Their review of 30 research studies highlighted that such partnerships create relationships and support that form the optimal environment for children’s learning and development. Altogether, these studies underscore the need for intentional, structured approaches to parent engagement that acknowledge the relational and emotional context of parent–school communication and actively work to transform adversarial dynamics into collaborative partnerships.

### 1.9. The Present Study

As outlined above, the evidence base for group-delivered mentalization interventions, while growing, remains emerging and methodologically heterogeneous, with most studies conducted in clinical rather than educational settings and few examining integrated multi-component interventions. The integration of psychoeducation, mentalization-based practice and peer support in a single group format is theoretically coherent: psychoeducation provides the knowledge base that enables mentalizing, while peer support offers the relational safety within which both learning and reflection can occur. Yet this specific combination has not been evaluated within alternative provision contexts where parent–school relationships may be particularly strained and where families are often disengaged from clinical pathways.

The present study seeks to address this gap by evaluating a weekly parent learning group delivered within an alternative provision school for children with neurodevelopmental conditions and conduct difficulties. The group integrates psychoeducational content concerning children’s neurodevelopmental profiles and behavioural presentations, mentalization-based principles including curiosity about the child’s and parent’s internal experience and strategies for reducing parental emotional dysregulation, and structured opportunities for peer support and shared reflection. A mixed-methods design was employed, combining pre- and post-intervention measures of parental reflective functioning and parental self-efficacy with semi-structured interviews exploring parents’ subjective experiences of the group.

The study aimed to investigate whether participation in the parent learning group was associated with changes in parental reflective functioning and parental self-efficacy, how parents experienced the group in terms of its impact on their wellbeing, knowledge and confidence, whether parents perceived changes in the quality of parent–child interactions following participation, and whether parents reported positive outcomes for their child that they attributed to their participation in the group. By situating this intervention within an alternative provision school and delivering it as part of a parent-school partnership model, the study also aimed to contribute to understanding how schools serving children with complex needs can more effectively engage and support parents as partners in their child’s education and development.

## 2. Methods

### 2.1. Research Design

This study employed a mixed-methods design, integrating quantitative measures of change with in-depth qualitative exploration of parents’ experiences. The quantitative component utilised a retrospective pretest–posttest design in which parents completed identical questionnaire batteries at a single time point, first reflecting retrospectively on the month before joining the group and then reflecting on their current experience after participation. This design was adopted as a pragmatic choice given the constraints of delivering research within an active school-based intervention where prospective baseline measurement was not feasible; however, it is important to note that retrospective pretest designs are known to introduce recall bias and may overstate change when participants are aware of desired outcomes [25]. The inclusion of a qualitative strand was therefore particularly important, as it provided an opportunity to triangulate quantitative findings and examine whether parents’ narrative accounts of change both confirmed and, in some cases, added nuance to the quantitative change scores. The qualitative component comprised semi-structured interviews exploring the mechanisms and processes through which parents experienced change, capturing the complexity and nuance of parents’ evolving understanding and skills. The integration of quantitative and qualitative data enabled a comprehensive understanding of both whether meaningful change occurred and how and why that change unfolded [26]. This approach is consistent with recommendations in the mixed-methods intervention evaluation literature, which emphasise that qualitative data can illuminate process mechanisms, contextual factors and participant experiences that quantitative measures alone cannot capture, thereby strengthening the interpretive framework for intervention studies with methodological limitations [26,27]. Ethical approval was obtained from the institutional ethics committee, and all participants provided written informed consent with assurances of confidentiality, anonymity and the right to withdraw at any time without consequence.

### 2.2. Participants and Setting

Twelve parents (mean age 38 years, range 28–52) were recruited from The Pears Family School, an alternative provision school in London serving children aged 5–11 with complex social, emotional and behavioural needs, including neurodevelopmental conditions and conduct difficulties. The school serves a socio-economically and culturally diverse community and operates from a systemic perspective that prioritises executive function development, stress management and social connectivity. Inclusion criteria required parents to have a child currently enrolled at the school, attend at least six weekly parent learning group sessions and consent to both the quantitative questionnaire and a semi-structured interview. All parents attending the group were invited to participate; no exclusion criteria were applied beyond the minimum attendance threshold.

Parents’ children ranged in age from 5 to 11 years and presented with a range of neurodevelopmental profiles and behavioural difficulties, including ADHD, autism spectrum conditions, conduct difficulties and mixed emotional and behavioural presentations. Participants represented diverse family structures, employment statuses and cultural backgrounds, reflecting the actual parent population of the school and ensuring that study findings represent the full range of family experiences within the school community.

The research was conducted within a school setting that adopts a whole-school approach to mental health and wellbeing, emphasising the active engagement of parents and carers. This approach views the child within their wider ecological context, recognising the role of family–school partnerships and differentiated levels of parental engagement (universal, targeted and specialist) in supporting pupils’ emotional, behavioural and mental health needs.

### 2.3. Intervention: Weekly Parent Learning Group

The weekly parent learning group ran throughout the school term, with each session lasting approximately 90 min. Sessions were facilitated by highly experienced practitioners specialising in systemic family work and mentalization-based approaches. The group integrated three core components consistent with the theoretical framework outlined above: psychoeducation, mentalization-based practice and structured peer support.

Psychoeducational content provided practical, accessible education on topics directly relevant to parents of children with neurodevelopmental and conduct difficulties. Content included understanding executive functions and their implications for behaviour, stress responses and their neurobiological basis, child development across middle childhood, common emotional and behavioural challenges associated with neurodevelopmental conditions, and strategies for emotion regulation. Material was delivered in accessible language with real-world examples, emphasising practical application to daily family life rather than abstract theory [12,14].

#### 2.3.1. Mentalization-Based Practice

Structured activities helped parents develop their capacity to “hold their child’s mind in mind”, considering their child’s perspective, emotional state and underlying needs rather than responding reactively to surface behaviours [28]. Parents practised perspective-taking through discussion of real family situations and reflection on their own interactions. Facilitators explicitly modelled curiosity about internal experience by asking questions such as “What might your child have been feeling in that moment?” and “What do you think was happening for them internally?” This component aimed to enhance parental reflective functioning—the capacity to understand one’s own and one’s child’s behaviour in terms of underlying mental states [16].

#### 2.3.2. Peer Support and Consultation

Dedicated time was allocated for parents to share experiences, problem-solve collaboratively and learn from one another’s strategies and insights. Facilitators actively encouraged peer-to-peer support and normalisation of shared challenges, addressing the social isolation frequently experienced by parents of children with complex needs [11] and leveraging the therapeutic benefits of shared experience documented in group intervention research [6].

#### 2.3.3. Facilitator Approach

Facilitators demonstrated mentalization principles through their interactions with parents by offering empathic attunement, genuine curiosity about emotional experiences and non-judgemental exploration of thoughts and feelings. This experiential modelling—“holding the parents’ minds”—was intended to provide parents with a lived experience of being mentalised, which they could then extend to their relationships with their children [28].

## 3. Measures

### 3.1. Quantitative Measures

Parents completed a questionnaire battery using a retrospective pretest–posttest format. For each item, parents first responded based on how they remembered feeling in the month before joining the group (retrospective pretest), and then based on how they felt at the time of completing the questionnaire, after participating in the group sessions (posttest). All items were rated on a 7-point Likert scale (1 = strongly disagree, 7 = strongly agree). Participants were informed that there were no right or wrong answers and that honest recall would help improve the programme for other parents.

#### 3.1.1. Parental Reflective Functioning Questionnaire (PRFQ)

The Parental Reflective Functioning Questionnaire [29] was used to assess parents’ capacity to reflect on their child’s and their own mental states. The PRFQ comprises 18 items rated on a 7-point scale, yielding three subscale scores. The Pre-Mentalizing (PM) subscale comprises items reflecting a non-mentalizing stance in which the parent attributes malicious intent or fails to consider the child’s mental states (e.g., “The only time I’m certain my child loves me is when he or she is smiling at me”; “Often, my child’s behaviour is too confusing to bother figuring out”). The Certainty about Mental States (CMS) subscale includes items reflecting excessive certainty about knowing the child’s mental states, indicating reduced openness to the opacity of mental experience (e.g., “I always know what my child wants”; “I can completely read my child’s mind”). The Interest and Curiosity in Mental States (IC) subscale comprises items reflecting genuine interest in understanding the child’s internal world (e.g., “I wonder a lot about what my child is thinking and feeling”; “I try to see situations through the eyes of my child”). The PRFQ demonstrates adequate reliability and validity as a brief measure of parental reflective functioning [29]. Lower scores on PM and CMS and higher scores on IC indicate more adaptive reflective functioning.

#### 3.1.2. Parenting Sense of Competence Scale (PSOC)

A 7-item adaptation of the Parenting Sense of Competence Scale [30,31] measured parental confidence, satisfaction and perceived ability to manage parenting challenges. Items assessed core dimensions of parenting self-efficacy and satisfaction, including statements such as “I am confident in my ability to meet my child’s needs” and “Being a parent gives me a strong sense of achievement”, interspersed with reverse-scored items (e.g., “I feel tense or anxious when parenting”). Higher scores indicate greater parenting self-efficacy and satisfaction. The PSOC has demonstrated strong psychometric properties across diverse parent populations [30].

#### 3.1.3. Memory Confidence Validity Check

A single-item validity check assessed participants’ confidence that their retrospective pretest responses accurately reflected their feelings at the earlier time point: “How confident are you that your responses in the retrospective pretest reflect your actual feelings at that earlier time?” Responses were rated on a 7-point scale (1 = not confident at all, 7 = very confident). This item was included to evaluate the reliability of the retrospective comparison and to identify participants whose retrospective responses might be unreliable.

#### 3.1.4. Accessibility Accommodations

To ensure inclusive participation, questionnaires were offered in both written and oral formats to accommodate parents with lower English literacy or reduced writing confidence. This accessibility measure ensured that language or literacy barriers did not prevent participation or compromise data quality, addressing a methodological gap in family engagement research where complex written instruments can inadvertently exclude parents from diverse linguistic backgrounds.

#### 3.1.5. Qualitative Data Collection: Semi-Structured Interviews

Semi-structured interviews lasting 45–60 min were conducted either in person at the school or via secure video call, according to participant preference. All interviews were audio-recorded with informed consent and transcribed verbatim.

An interview guide was developed based on the study’s theoretical framework and research aims, organised around six thematic domains. Motivation and overall experience explored what motivated parents to join, how their experience evolved across the weeks, whether initial expectations matched reality, and how the group influenced their sense of belonging or connection with other parents and the school community, with an opening question (“Can you tell me a bit about what a typical day looks like with your child?”) used to establish rapport and contextualise subsequent discussion. Understanding and applying mentalization principles examined how parents described their capacity to “hold their child’s mind in mind” compared with when they began, how their ability to consider their child’s thoughts and feelings developed during the sessions, and how psychoeducational content enhanced their understanding of their child. Affect regulation and emotional changes explored how parents’ ability to manage emotions during parenting challenges developed during the programme, elicited concrete examples of applying emotion regulation skills and investigated changes in stress levels or emotional responses since joining. Parent–child relationship evolution focused on how interactions with their child evolved over the course of the programme and whether parents noticed changes in their child’s emotional reactions or behaviours, including moments when the group experience helped them connect more effectively with their child. Home–school engagement development explored how communication or collaboration with school staff changed since joining the group and elicited specific examples of applying group learning in school-related situations. Perceived child outcomes and group process probed changes noticed in the child’s school engagement or wellbeing, the value of peer support and consultation, the most beneficial aspects of the group, key benefits gained, suggestions for future groups, particularly meaningful components, and how parents hoped to use their learning going forward.

Interviews were conducted in a manner consistent with mentalization principles, with genuine curiosity, a non-judgmental stance and interest in the parent’s internal experience and perspective. Interviewers used open-ended prompts and reflective follow-up questions to elicit detailed accounts of parents’ experiences.

## 4. Data Analysis

### 4.1. Quantitative Analysis

Descriptive statistics (means, standard deviations) were computed for all measures at both time points. Paired-samples *t*-tests compared retrospective pretest and posttest scores on the PRFQ subscales (Pre-Mentalizing, Certainty about Mental States, Interest and Curiosity in Mental States) and the PSOC total score. Cohen’s *d* effect sizes quantified the magnitude of pre-to-post changes. Given the small sample size, non-parametric Wilcoxon signed-rank tests were also conducted to verify parametric results. Alpha was set at 0.05 for all analyses.

### 4.2. Qualitative Analysis

Interview transcripts underwent reflexive thematic analysis following the six-phase approach described by Braun and Clarke [32,33]. Analysis employed both deductive and inductive coding strategies. Initial deductive codes were derived from the study’s theoretical framework, including constructs from mentalization theory (reflective functioning, pre-mentalizing, affect regulation, interest in mental states), psychoeducational intervention research (knowledge acquisition, skill development) and systemic practice (executive functions, stress management, social connectivity, peer learning, home-school collaboration). Inductive coding captured emergent themes reflecting parents’ subjective experience of change and unanticipated programme impacts.

### 4.3. Integration of Quantitative and Qualitative Data

Quantitative and qualitative findings were integrated at the interpretation stage following a convergent design [26]. Qualitative themes were mapped onto quantitative constructs to examine whether parents’ narrative accounts of change were consistent with measured improvements in reflective functioning and parenting self-efficacy. Points of convergence and divergence between data sources were identified and discussed.

## 5. Results

### 5.1. Sample

Twelve parents (*N* = 12) completed the retrospective pretest–posttest questionnaire battery following participation in the weekly parent learning group. All participants reported high confidence that their retrospective pretest responses accurately reflected their feelings prior to joining the group (M = 5.67, SD = 0.99 on a 7-point scale), supporting the validity of the retrospective comparison.

### 5.2. Quantitative Results

Parents reported a significant decrease in pre-mentalizing from retrospective pretest (M = 4.47, SD = 2.47) to posttest (M = 1.53, SD = 0.73), *t*(11) = 4.63, *p* < 0.001, *d* = 1.34, indicating substantial movement away from attributing malicious intent or failing to consider their child’s mental states. The Wilcoxon signed-rank test confirmed this finding (W = 0, *p* < 0.002).

Parents showed a decrease in excessive certainty about their child’s mental states from pretest (M = 4.06, SD = 1.38) to posttest (M = 3.00, SD = 0.67), though this difference approached, but did not reach, conventional significance, *t*(11) = 2.17, *p* = 0.053, *d* = 0.63. The Wilcoxon signed-rank test yielded a comparable result (W = 14, *p* = 0.05). The medium effect size suggests a trend towards greater openness to the opacity of children’s mental experience, with some individual variability.

Parents demonstrated a significant increase in interest and curiosity about their child’s mental states from pretest (M = 4.67, SD = 1.01) to posttest (M = 6.47, SD = 0.23), *t*(11) = 6.33, *p* < 0.001, *d* = 1.83. The Wilcoxon signed-rank test confirmed this result (W = 0, *p* < 0.001).

Parents also reported a significant increase in parenting self-efficacy and satisfaction on the PSOC from pretest (M = 2.84, SD = 1.15) to posttest (M = 5.19, SD = 0.76), *t*(11) = 5.57, *p* < 0.001, *d* = 1.61, reflecting substantial improvements in confidence, perceived ability to manage challenges and satisfaction in the parenting role (Wilcoxon signed-rank test, W = 0, *p* < 0.001).

**Figure 1:**
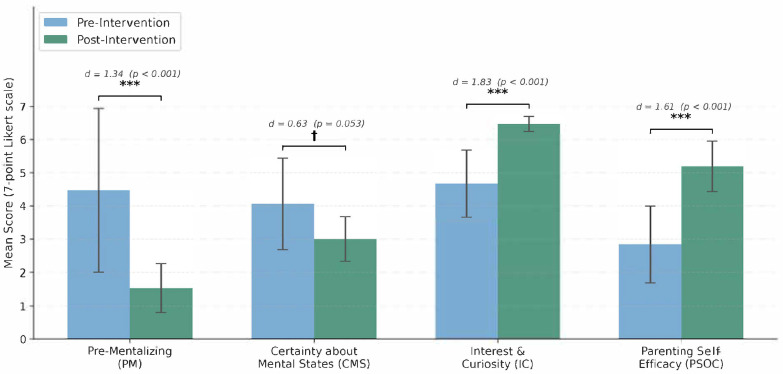
Pre- and post-intervention mean scores on the Parental Reflective Functioning Questionnaire subscales—Pre-Mentalizing (PM), Certainty about Mental States (CMS), and Interest and Curiosity in Mental States (IC)—and the Parenting Sense of Competence Scale (PSOC). All items were rated on a 7-point Likert scale. Lower PM and CMS scores and higher IC score reflect more reflective parenting. *** *p* < 0.001. † Error bars represent standard deviations (SD).

It is important to note an asymmetry in standard deviations between retrospective pretest and posttest scores, particularly evident for the Pre-Mentalizing subscale (pretest SD = 2.47 versus posttest SD = 0.73) and the Parenting Sense of Competence scale (pretest SD = 1.15 versus posttest SD = 0.76). This substantial reduction in variance at posttest may reflect a floor or ceiling effect, whereby participants converge towards more adaptive scores following intervention, leaving reduced variability at posttest. This SD asymmetry has implications for the interpretation of effect sizes: Cohen’s *d* values computed using the pretest SD as the denominator may be inflated relative to those computed using pooled standard deviations, and readers should interpret the large effect sizes reported here with this caveat in mind. The convergent non-parametric Wilcoxon signed-rank tests provide additional support for the reliability of these findings. Changes between pre- and post-intervention scores across parental mentalizing and parenting outcomes are presented in Figure 1.

Overall, the quantitative findings indicate that participation in the parent learning group was associated with large improvements in parental reflective functioning (reduced pre-mentalizing, increased interest and curiosity) and parenting self-efficacy, with a trend towards reduced excessive certainty about children’s mental states.

### 5.3. Qualitative Results

Reflexive thematic analysis of interview responses yielded six interconnected themes reflecting parents’ experiences of change through participation in the group.

#### 5.3.1. Theme 1: A Safe, Non-Judgemental Space for Authentic Self-Expression

The most consistently reported experience was the creation of a psychologically safe environment in which parents could express themselves without fear of judgment. Within the framework of mentalization theory, this finding is significant because mentalizing is understood to be a capacity that is highly sensitive to relational context—it flourishes in conditions of epistemic trust and safety but is inhibited by perceived threat, judgment or social evaluation [34]. The group environment thus appears to have functioned as a precondition for the activation and development of reflective capacities, rather than merely a pleasant social experience. The safety reported by parents can be understood as enabling the establishment of epistemic trust—the willingness to regard interpersonal communication as relevant, trustworthy and generalisable to other contexts [35]—which in turn created conditions under which new learning about parenting and mentalizing could take root.

One parent described this space as “*No* *one overtly says it, but you can really lower your defences and just be people looking for a way forward together*”, and another said “*The sense of safety and lack of judgment was extremely meaningful to me. This group helped me feel like a good parent, not a failing one*”. Parents reflected that “*I didn’t know what to expect, but I didn’t think it would be as supportive, safe or positive as it has been. Hearing from others and feeling there are people who understand is really helpful…”*, and “*I feel so much more understood and supported when spending time with other parents who get my situation. There is no judgement or blame. You can be honest and feel more relaxed*”.

Parents described this safety as qualitatively different from other school or parenting contexts: “*The combination of warmth and tentativeness made me feel very comfortable. People are kind and also everyone has their own level of openness on any given day and that is reassuring--you can be how you feel, not what is ‘expected’, like the school gate always was*”. For parents with their own neurodevelopmental conditions, this safety carried additional significance: “*I have been diagnosed as having ADHD since we joined, and I feel for the first time that I can be my actual self there...no masking. I don’t feel like I have to pretend to be together, or know what I’m doing, or get things, or make small talk if I’m drained*”. The experience of shared struggle and mutual recognition was central: “*Realising I am not alone in these experiences was very strengthening... It created a feeling of shared experience rather than isolation*”, and “*I feel so much connection and warmth--we are all in this, we all struggle to be the kind of parents we want to be, we can be honest about that and not feel judged. Fundamentally, the group makes me feel capable and not alone*”. This pattern is consistent with research demonstrating that mentalization gains in group settings are frequently mediated by group processes—including relational safety, peer identification and shared vulnerability—rather than by didactic content alone [19]. The group context appears to have reduced epistemic hypervigilance, enabling parents to move from defensive self-presentation to authentic reflective engagement. Mentalization-based group therapy literature similarly emphasises that therapeutic change often emerges through group relational processes rather than solely through instructional elements.

#### 5.3.2. Theme 2: Enhanced Mentalizing Capacity

Parents described meaningful shifts in their ability to consider their child’s internal world, moving from reactive responses to surface behaviour toward curiosity about underlying mental states. Theoretically, this shift can be understood as a movement along the dimension of parental reflective functioning (PRF)—from pre-mentalizing modes characterised by teleological thinking and psychic equivalence toward more genuinely mentalizing stances marked by curiosity, perspective-taking and recognition of the opacity of mental states [29,36]. The qualitative data thus illuminate the process by which quantitative improvements in PRF manifested in everyday parenting interactions.

One parent reflected “*I feel I am better at being mindful of how my child might be feeling and exploring this with him before jumping to conclusions or making judgments, while trying to remain more positive”.* Another parent said “*I am more able to pause and consider what my child might be feeling or thinking in difficult moments. I’ve learned to look beyond the behaviour and think about what might be driving it*”. Hearing other parents’ children’s stories helped provide context: “*Hearing other people’s children’s stories gives a lot of context for understanding my own daughter. I listen to people’s stories and thoughts and there’s enough distance from my own situation to then connect a few dots in our own experience and see things as she might*”.

Parents articulated “*holding the child’s mind in mind*” as an active process: “*See things as necessary to her, not a choice, and accommodate her needs in this context. I always think of the line ‘all behaviour is communication’ but understand it much better now*”. Another parent shared “*Trying to understand what my child might be experiencing internally, not just what I see externally. Remembering that there are feelings and thoughts behind the behaviour*”. A particularly vivid example illustrated how group learning translated into mentalizing insight: “*One day my daughter was in very strong form, very happy to be going to school… As we got into the building and were climbing the stairs, she slowed and wanted to start a conversation about one of her special interests, Owlhouse. They can be hour-long one-sided explanations, and we were a few staircases away from the group getting ready to go to Music, so I dissuaded her from talking about it due to time, as we were almost at our floor. She crumpled on the spot and couldn’t climb further... Only later this day in the parents group I realised, hearing [the facilitator] talking to us, that this conversation—Owlhouse—was a safe space she could take refuge in because she was feeling very daunted and overwhelmed, and that kids have many ways of communicating what they need which is not always what it seems to be*”. This account is particularly instructive from a mechanistic perspective: it illustrates how facilitator-led group discussion (the process mechanism) enabled a shift from teleological interpretation of the child’s behaviour—viewing it as obstructive delay—toward a mentalizing interpretation that recognised the child’s bid for emotional regulation through a familiar special interest. The parent’s insight did not arise from didactic instruction but from the facilitator’s modelling of curiosity in the group context, which the parent then applied retrospectively to her own experience. This is consistent with theoretical propositions that mentalization is transmitted experientially through relational processes rather than through explicit teaching alone [34].

#### 5.3.3. Theme 3: Improved Affect Regulation and Reduced Parental Stress

Parents reported meaningful changes in their capacity to manage their own emotions during challenging parenting moments, describing a shift from reactivity to reflective responding: “*I am more able to pause and regulate my emotions before reacting. I feel more aware of my emotional responses and better able to manage them*”. Within mentalization theory, this shift is understood as reflecting enhanced mentalised affectivity—the capacity to experience, identify and regulate emotions while maintaining a reflective stance toward one’s own internal states [37]. Parental affect dysregulation is a hallmark of pre-mentalizing states and is closely linked to coercive or withdrawn parenting patterns; the capacity to pause, reflect and regulate before responding represents a core mechanism through which improved parental mentalizing translates into more adaptive caregiving behaviour [38,39]. Increased patience was noted: “*More patience and more waiting to see rather than jumping in to ‘fix’ things is helping us both*”, and one parent observed “*I am much less prone to feeling stressed during the school day, as I know he is in a safe place. I also try to be more patient when challenges arise”.* The reduction in stress was described not merely as lower arousal but as a fundamental shift in the mental labour of parenting: “*I can be myself for the first time in an educational/school gate environment and god it is so nice to use my mental energy to just be, rather than be always working out what that should look like. So, very much lower waste of mental energy, leaving me with more for my family life and self*”. This account suggests that improvements in affect regulation were not limited to discrete parenting episodes but extended to a broader reduction in the chronic hypervigilance and impression management that characterises parents who have experienced judgment or stigmatisation—a pattern consistent with the relaxation of epistemic hypervigilance theorised to occur when epistemic trust is established [35]. Parents also reported reductions in guilt and self-criticism: “*I feel less reactive and more grounded. I experience less guilt and self-criticism as a parent*”, and “*I am still working on managing my emotions during challenging moments, but I have become better at reflecting afterward and thinking about how I can respond more effectively next time*”. Research on mentalization similarly suggests that the capacity to reflect on internal states plays a central role in emotion regulation processes and adaptive interpersonal functioning.

#### 5.3.4. Theme 4: Transformed Parent–Child Interactions

Parents described qualitative shifts in the nature of their interactions with their children, characterised by increased patience, attentiveness and relational connection: “*Our interactions feel calmer and more connected. I am more patient and attentive in our daily interactions*”. For some, transformation involved a fundamental reframing of parenting priorities from rule enforcement to emotional safety: “*Understanding that emotional and physical safety is what matters most and if that is there then it really doesn’t matter if she wants to eat dinner on the sofa or play Animal Crossing all morning, and we can just relax a bit in the rules, feeling equilibrium matters much more*”, and “*This has had a greater impact on my partner who is also part of the group as he and our child now spend more time together and have a better understanding of each other, which has led to more freedom for me*”. Parents also reported observable changes in their children’s responses: “She’s way more relaxed, is loving having stimuli like art and the park and absolutely loves having people to talk to, and all this makes her feel a little more able to share her feelings than she used to”, and “*My child seems calmer and more regulated at times. There are fewer escalations, or they pass more quickly*”. These accounts suggest the emergence of positive relational reciprocity—a bidirectional process in which improved parental mentalizing elicits more regulated child behaviour, which in turn reinforces parental confidence and further mentalizing, consistent with theoretical models of the intergenerational transmission of mentalizing capacity [36,40]. In a particulary striking account of change, one parent described their child’s transformation: “*She goes to school! She has effectively had no education for 16 months... and we were housebound; she could only exist in the safe space of home... Now she is enthusiastic, future-looking, wants to go to school, pushes through harder situations instead of retreating, is much less regressive, is proud of her achievements, is engaged more with others, and feels emotionally safe, understood and appreciated at school*”.

#### 5.3.5. Theme 5: Psychoeducational Understanding as a Foundation for Change

Parents described how psychoeducational content—particularly around executive functioning, stress responses and neurodevelopmental conditions—provided a conceptual framework that reframed their understanding of their child’s behaviour: “*It helped me understand how stress and regulation affect my child’s behaviour. I better understand my child’s difficulties as developmental and emotional, not intentional*”. From a theoretical perspective, this cognitive reframing can be understood as a shift away from teleological reasoning—in which behaviour is interpreted solely in terms of observable actions and their apparent intentionality—toward a mentalizing stance that recognises the developmental and neurobiological underpinnings of the child’s experience. Psychoeducation thus appears to have functioned as a cognitive bridge to mentalization, providing the conceptual scaffolding necessary for parents to move from frustration-driven attributions of deliberate misbehaviour to curiosity about the child’s internal regulatory capacities. One parent noted “*Still ongoing. So far just the reassurance that her way of being often has an explanation. Also I am ND also, so there’s a double effect of helping both of us get what’s going on in our heads and why*”. This understanding functioned as a bridge to mentalizing, replacing attributions of intentional misbehaviour with developmental explanations: “*Learning about executive function helped me adjust my expectations. It gave me language and tools to better understand what my child is going through*”.

#### 5.3.6. Theme 6: Facilitation Style and Group Process as Active Ingredients

Parents identified specific features of the group process and facilitation style as particularly meaningful, distinguishing this experience from other forms of support: “*B and N are very gentle and make you feel like you can say whatever is on your mind, but they also subtly direct the conversation into themes and adapt to what we are saying*”. This finding is consistent with research demonstrating that mentalization gains are often mediated by the quality of group processes and facilitator modelling rather than by programme content alone [19]. The facilitators’ approach—characterised by curiosity, empathic attunement and non-judgemental exploration—can be understood as directly modelling the mentalizing stance that the intervention aimed to develop in parents. By “holding the parents’ minds”, facilitators provided an experiential template for reflective parenting: parents first experienced being mentalised, and then extended this relational capacity to their interactions with their children. This process aligns with theoretical models of epistemic trust [34], in which the therapist’s or facilitator’s genuine interest in the individual’s internal experience opens a channel for learning that is resistant to purely didactic transmission.

The facilitators’ approach was described as modelling the very mentalizing stance parents were developing: “*Instead of saying ‘no, no, you’re a great parent’, [the facilitators] opened it out to us so we could all talk about that feeling like a bad parent and how that works in our heads, and the detrimental effect of it, and guided us as we all pushed each other upward and used our shared experience*”. Specific tools, such as reflective cards, were identified as facilitating authentic sharing: “*The cards are a great way to connect with the other people’s stories and experiences. They are a prop, which lets us say whatever is on our minds that week without feeling egocentric or like you’re taking up too much space. They make the room feel equal and open*”. The emotional power of peer sharing was captured poignantly: “*One person said she felt ‘trapped’ one week and began crying, and it felt both like she’d been scared to say that out loud for fear of judgment and was freer now for saying it, and that she’d given the room a bit of a gift, because lots of people seemed to feel very similarly but invisibly”.* A summary of the qualitative themes is presented in Table 1.

While the six themes reflected patterns across most participants, the analysis also captured more nuanced and divergent experiences. Some parents described change as ongoing and incremental rather than complete, and one parent experienced the psychoeducational content as personally meaningful following their own ADHD diagnosis. These variations were integrated into the thematic analysis, with no predominantly negative or contradictory accounts reported; themes were broadly consistent, differing mainly in depth and pace of change.

**Table 1:** Summary of Qualitative Themes and Representative Quotations.

Theme	Description	Representative Quotations
**1. Safe, Non-Judgmental Space for Authentic Self-Expression**	Creation of a psychologically safe environment enabling parents to express themselves without fear of judgment, share feelings of vulnerability, and experience mutual recognition of shared struggles.	*“No one overtly says it, but you can really lower your defences and just be people looking for a way forward together.”* *“I didn’t know what to expect, but I didn’t think it would be as supportive, safe or positive as it has been. Hearing from others and feeling there are people who understand is really helpful…”* *“I feel so much more understood and supported when spending time with other parents who get my situation. There is no judgement or blame. You can be honest and feel more relaxed.”* *“The sense of safety and lack of judgment was extremely meaningful to me. This group helped me feel like a good parent, not a failing one.”* *“I have been diagnosed as having ADHD since we joined, and I feel for the first time that I can be my actual self there...no masking.”* *“Realising I am not alone in these experiences was very strengthening... It created a feeling of shared experience rather than isolation.”*
**2. Enhanced Mentalizing Capacity**	Meaningful shifts in the ability to consider the child’s internal world, moving from reactive responses to surface behaviour toward curiosity about underlying mental states.	*“I feel I am better at being mindful of how my child might be feeling and exploring this with him before jumping to conclusions or making judgments, while trying to remain more positive.”* *“I am more able to pause and consider what my child might be feeling or thinking in difficult moments. I’ve learned to look beyond the behaviour and think about what might be driving it.”* *“Hearing other people’s children’s stories gives a lot of context for understanding my own daughter... see things as she might.”* *“I always think of the line ‘all behaviour is communication’ but understand it much better now.”* *“Kids have many ways of communicating what they need, which is not always what it seems to be.”*
**3. Improved Affect Regulation and Reduced Parental Stress**	Enhanced capacity to manage emotions during challenging parenting moments, shift from reactivity to reflective responding, and a reduction in the mental labour of parenting.	*“I am more able to pause and regulate my emotions before reacting. I feel more aware of my emotional responses and better able to manage them.”* *“More patience and more waiting to see rather than jumping in to ‘fix’ things is helping us both.”* *“I am much less prone to feeling stressed during the school day, as I know he is in a safe place. I also try to be more patient when challenges arise.”* *“I can be myself for the first time in an educational/school gate environment... very much lower waste of mental energy, leaving me with more for my family life and self.”* *“I feel less reactive and more grounded. I experience less guilt and self-criticism as a parent.”* *“I am still working on managing my emotions during challenging moments, but I have become better at reflecting afterward and thinking about how I can respond more effectively next time.”*
**4. Transformed Parent–Child** **Interactions**	Qualitative shifts in the nature of interactions with children, characterised by increased patience, attentiveness, relational connection, and observable changes in children’s responses.	*“Our interactions feel calmer and more connected. I am more patient and attentive in our daily interactions.”* *“Understanding that emotional and physical safety is what matters most... we can just relax a bit in the rules, feeling equilibrium matters much more.”* *“This has had a greater impact on my partner, who is also part of the group, as he and our child now spend more time together and have a better understanding of each other, which has led to more freedom for me.”* *“My child seems calmer and more regulated at times. There are fewer escalations, or they pass more quickly.”* *“She goes to school! She has effectively had no education for 16 months... Now she is enthusiastic, future-looking, wants to go to school, pushes through harder situations instead of retreating.”*
**5. Psychoeducational** **Understanding as Foundation for Change**	Psychoeducational content (executive functioning, stress responses, neurodevelopmental conditions) provided a conceptual framework reframing understanding of the child’s behaviour from intentional to developmental.	*“It helped me understand how stress and regulation affect my child’s behaviour. I better understand my child’s difficulties as developmental and emotional, not intentional.”* *“Still ongoing. So far, just the reassurance that her way of being often has an explanation. Also, I am ND also, so there’s a double effect of helping both of us get what’s going on in our heads and why.”* *“Learning about executive function helped me adjust my expectations. It gave me language and tools to better understand what my child is going through.”*
**6. Facilitation Style and Group Process as Active Ingredients**	Specific features of group process and facilitation style identified as meaningful: facilitators modelling mentalizing stance, use of reflective tools, and emotional power of peer sharing.	*“[Facilitators] are very gentle and make you feel like you can say whatever is on your mind, but they also subtly direct the conversation into themes and adapt to what we are saying.”* *“Instead of saying ‘no, no, you’re a great parent’, [the facilitators] opened it out to us so we could all talk about that feeling like a bad parent... and guided us as we all pushed each other upward.”* *“The cards are a great way to connect with other people’s stories and experiences. They are a prop, which lets us say whatever is on our minds that week without feeling egocentric.”* *“One person said she felt ‘trapped’ one week and began crying... she’d given the room a bit of a gift, because lots of people seemed to feel very similarly but invisibly.”*

Note. Themes derived from reflexive thematic analysis of semi-structured interviews with parents (N = 12) following participation in at least 6 sessions of a weekly parent learning group.

### 5.4. Integration of Quantitative and Qualitative Findings

Quantitative and qualitative findings converged in several important respects, demonstrating coherence between measured change and parents’ narrative descriptions of their experience. This convergence is consistent with best practice in mixed-methods intervention evaluation, where the integration of quantitative outcome data with qualitative process data can strengthen interpretive claims, identify mechanisms of change not captured by standardised measures, and reveal the contextual conditions under which intervention effects operate [26,27].

The significant reduction in pre-mentalizing scores (*d* = 1.34) was echoed in qualitative accounts of parents moving from reactive, attribution-based responding to curious, reflective engagement with their children’s internal experiences. The large increase in interest and curiosity about children’s mental states (*d* = 1.83) aligned with parents’ descriptions of actively seeking to understand their child’s perspective and recognising that “all behaviour is communication”. The substantial improvement in parenting self-efficacy (*d* = 1.61) was mirrored in parents’ accounts of feeling “*capable and not alone*,” experiencing reduced guilt and self-criticism, and developing practical tools for managing parenting challenges.

Importantly, the qualitative data did not merely confirm the quantitative findings but also extended and nuanced them. In particular, the qualitative strand illuminated process mechanisms not captured by the PRFQ or PSOC—including the role of epistemic trust established through facilitation style, the function of peer identification in reducing epistemic hypervigilance, and the way psychoeducational content served as a cognitive bridge to mentalizing rather than operating as a separate intervention component.

The borderline finding for certainty about mental states (*p* = 0.053, *d* = 0.63) gains nuance when viewed alongside the qualitative data, which suggest that some parents developed appropriate confidence in understanding their child (adaptive CMS increase) while simultaneously maintaining openness to epistemic uncertainty about their child’s internal world. This pattern—where reduced excessive certainty coexists with increased appropriate understanding—may explain the smaller and less uniform quantitative change on this subscale.

It is also important to note that the qualitative data revealed instances where parents’ accounts added complexity to the quantitative picture rather than simply corroborating it. For example, while the PSOC scores indicated uniformly improved self-efficacy, several parents described self-efficacy gains as uneven and context-dependent—feeling more confident in some situations while recognising continued vulnerability in others. Similarly, while the PRFQ captured broad improvements in mentalizing stance, parents’ narratives revealed that mentalizing capacity fluctuated with stress levels and was experienced as an ongoing developmental process rather than a stable trait change. These nuances, accessible only through qualitative inquiry, underscore the value of mixed-methods approaches in intervention evaluation and caution against interpreting quantitative effect sizes as reflecting uniform, stable change across all participants and contexts.

Overall, the integration of findings suggests that the parent learning group facilitated change through multiple interconnected pathways. Psychoeducational content provided a cognitive framework for understanding children’s behaviour, the mentalizing stance modelled by facilitators offered an experiential template for reflective parenting, and the safe, non-judgemental group environment reduced isolation and enabled authentic engagement with difficult parenting experiences. The convergence—and, equally importantly, the productive divergence—between quantitative and qualitative data strengthens the interpretive framework and exemplifies the value of mixed-methods designs for intervention research, particularly when quantitative methods are limited by small sample sizes, retrospective designs and brief self-report measures.

It is important to distinguish findings supported by both quantitative and qualitative data from those emerging mainly from qualitative accounts. Improvements in pre-mentalizing, interest in mental states, and parenting self-efficacy are supported by both, providing the strongest evidence of change. In contrast, enhanced parent–school partnerships, perceived child outcomes, and broader relational changes (Themes 3 and 4) arise mainly from qualitative data and are not captured by PRFQ or PSOC, offering valuable insights for hypothesis generation but based on a less robust evidential foundation.

## 6. Discussion

### 6.1. Summary of Findings

This study evaluated an integrated parent learning group combining psychoeducation, mentalization-based practice and peer support within an alternative provision school. The convergent mixed-methods design yielded preliminary evidence of meaningful improvements in parental reflective functioning and self-efficacy, while qualitative findings illuminated the process mechanisms through which change occurred.

Quantitatively, large effect sizes were observed for reduced pre-mentalizing (*d* = 1.34), increased interest and curiosity in children’s mental states (*d* = 1.83), and improved parenting self-efficacy (*d* = 1.61), with a trend toward reduced excessive certainty (*d* = 0.63, *p* = 0.053). Qualitative analysis identified six interconnected themes—relational safety, enhanced mentalizing, improved affect regulation, transformed parent–child interactions, psychoeducational understanding and facilitative group processes—that converged with and enriched the quantitative findings.

### 6.2. Parental Reflective Functioning

The improvements in parental reflective functioning observed in the present study are consistent with and extend outcomes reported in existing mentalization-based parenting interventions. However, they must be interpreted within the context of an emerging evidence base characterised by methodological heterogeneity and a predominance of small, uncontrolled studies [1,5].

The pattern of reduced pre-mentalizing and increased interest and curiosity mirrors outcomes from mentalization-oriented group programmes such as the Lighthouse Parenting Program [18]. Importantly, however, the qualitative data add mechanistic depth to the quantitative findings. Parents’ descriptions of moving from reactive, attribution-based responding to curious engagement with their child’s internal world illustrate the process by which improved PRF manifests in everyday interactions—a process that standardised measures like the PRFQ, being brief and self-report in nature, can capture only in aggregate. The qualitative strand revealed that this shift was not experienced as a discrete skill acquisition but as a gradual transformation in relational orientation, facilitated by the interaction of psychoeducational understanding, facilitator modelling and peer reflective processes.

These findings reinforce conclusions from narrative reviews suggesting that PRF is associated with more adequate caregiving and improved child outcomes [38,41] and extend this literature by indicating that pre-mentalizing can be modified through a brief group intervention even among parents facing the chronic stressors of caring for children with complex educational and behavioural needs. The integration of quantitative and qualitative evidence suggests that change in PRF was not solely a function of intervention content but wasdepended critically dependent on the relational and group-process conditions—epistemic trust, relational safety, and facilitator modelling—within which that content was delivered.

The magnitude of improvement on the Interest and Curiosity subscale (*d* = 1.83) is particularly noteworthy and aligns with theoretical propositions that the capacity to “hold the child’s mind in mind” represents a core mechanism through which mentalizing supports adaptive parenting practice [40]. The qualitative data, in which parents described new capacities to pause and consider what their child might be feeling and to recognise that “all behaviour is communication”, provide empirical support for this claim by showing how enhanced curiosity became embedded in everyday parenting interactions.

The significant reduction in pre-mentalizing observed in this study (*d* = 1.34) also reinforces conclusions from narrative reviews suggesting that higher parental reflective functioning is associated with more adequate caregiving and improved child outcomes, while lower reflective functioning is linked to greater child emotional and behavioural problems [38]. Extending this literature, the present findings suggest that pre-mentalizing—the tendency to attribute malicious intent or to abandon attempts to understand the child’s experience—can be modified through a brief group intervention even among parents facing the chronic stressors associated with caring for children with complex educational and behavioural needs.

The borderline finding for Certainty about Mental States (*d* = 0.63, *p* = 0.053) in this study warrants interpretation in light of recent conceptualisations that mentalization is dynamic and relationally specific, and that different dimensions of reflective functioning may respond differently to intervention. Qualitative data from this study suggest that some parents developed appropriate confidence in understanding their child while simultaneously maintaining openness to not-knowing—a pattern that reflects adaptive epistemic flexibility rather than rigid certainty. This nuanced picture, where increased appropriate understanding coexists with openness to ambiguity, suggests that future research should use measures designed to distinguish adaptive certainty grounded in attunement from maladaptive certainty reflecting assumptions or projection.

### 6.3. Parenting Self-Efficacy

The improvement in parenting self-efficacy aligns with evidence that multi-component programmes combining psychoeducation with emotional and relational support produce stronger effects than education alone [13]. Theoretically, the gains in self-efficacy observed here can be understood through Bandura’s [42] framework as arising from multiple sources: mastery experiences (applying new skills at home), vicarious experience (observing peers managing similar challenges), social persuasion (receiving validation from facilitators and peers), and reduced physiological arousal (lower stress and reactivity). The integration of mentalization-based practice may have enhanced these self-efficacy pathways by enabling parents to interpret challenging child behaviour as communicative rather than provocative, thereby reducing the experience of parenting failure that erodes self-efficacy over time.

Importantly, our study extends this broader evidence base to parents of school-age children with neurodevelopmental and conduct difficulties in alternative provision settings—a group that has received limited prior attention in intervention research. Parents’ qualitative accounts emphasise that self-efficacy gains were not merely cognitive but deeply relational—emerging from the experience of being understood, validated and recognised as a competent parent within a non-judgemental group context. This relational dimension of self-efficacy gains has implications for intervention design: it suggests that programmes aiming to enhance parental confidence should attend not only to skill transmission but to the creation of conditions that support epistemic trust and reduce the shame and self-blame that frequently characterise parents of children with complex difficulties.

### 6.4. The Role of Psychoeducation

A distinctive feature of our intervention was its integration of psychoeducational content with mentalization-based practice. Parents consistently reported that learning about executive functions, stress responses and neurodevelopmental profiles provided a conceptual framework that reframed attributions of misbehaviour as developmental rather than intentional. Within the theoretical framework of this study, psychoeducation appears to have functioned not as a separate intervention component but as a cognitive scaffold for mentalization, reducing the ambiguity and frustration that maintain pre-mentalizing stances and creating conditions under which reflective curiosity about the child’s internal world could develop. This mechanism is consistent with models proposing that epistemic trust—the precondition for receptivity to new learning—can be established through the provision of information that is experienced as genuinely relevant and explanatory, thereby opening a communicative channel through which more relational and reflective learning can occur [35].

Whereas prior research has typically examined psychoeducation and mentalization-based approaches as separate modalities, our findings suggest that their integration within a single group format may produce synergistic effects, with psychoeducation providing the conceptual grounding that renders children’s behaviour interpretable, and mentalization-based practice providing the relational and reflective skills to act on that understanding. This observation generates a testable hypothesis: that psychoeducation functions as a cognitive bridge to mentalization, and that this bridge may be especially valuable for parents whose children’s behaviour has previously seemed incomprehensible or deliberately provocative.

### 6.5. Peer Support and Group Process

The qualitative findings identify peer support as a critical process mechanism, consistent with evidence that group contexts can reduce parental isolation and facilitate emotional support [43]. Theoretically, the group’s role can be understood through the lens of epistemic trust: the experience of being understood by others who share similar struggles appears to have reduced epistemic hypervigilance—the defensive closure to new information that characterises individuals who have experienced repeated judgment or social exclusion—thereby creating conditions under which new learning about parenting and mentalizing could be received and integrated.

Descriptions of the group as a space where parents could “lower defences and just be people looking for a way forward together” resonate with characterisations of peer support as providing deeply felt empathy, encouragement and mutual assistance, and suggest that such mechanisms can be successfully harnessed in school-based parent groups. The significance of facilitation style in our study warrants particular emphasis. Research on mentalization-based group interventions has increasingly recognised that mentalization gains are often mediated by the quality of group processes and facilitator stance rather than by programme content alone [19]. The facilitators’ modelling of curiosity, empathic attunement and non-judgemental exploration—described by parents as qualitatively different from other professional interactions—appears to have provided an experiential template for reflective parenting. This is consistent with the theoretical proposition that mentalizing is transmitted through relational experience rather than didactic instruction, and that the facilitator’s capacity to “hold the parents’ minds” functions as a prerequisite for parents’ developing capacity to “hold the child’s mind in mind” [34].

Interpreted through the lens of MBT-G [19], the safe, non-judgmental environment described in Theme 1 reflects the emergence of a mentalizing group culture—a relational context in which epistemic trust could be established not only between individual parents and facilitators, but across the group as a whole. Yalom and Leszcz’s [20] therapeutic factors are clearly discernible in parents’ accounts: universality (recognising that one’s struggles are shared), group cohesion (a deep sense of belonging and mutual acceptance), altruism (the meaningful experience of contributing to others’ progress), and instillation of hope (witnessing peers’ change). These group-specific mechanisms may have amplified individual gains beyond what could be achieved through individual or dyadic formats alone. The facilitators’ role described in Theme 6 corresponds closely to the MBT-G concept of the group therapist as a mentalizing model who actively cultivates the group’s reflective culture—not merely by teaching mentalizing as a skill but by embodying epistemic curiosity in moment-to-moment responses, and by shaping the group norm towards curious, non-judgmental exploration of internal experience [19].

### 6.6. School-Based Delivery and Parent-School Partnership

The delivery of this intervention within an alternative provision school extends the evidence base for mentalization-based parent interventions beyond the clinical contexts in which they have predominantly been evaluated. While systematic reviews have identified promising effects of school-based mentalization interventions on student socio-emotional competencies [24], the present study is, to our knowledge, among the first to evaluate a mentalization-oriented parent group embedded within an alternative provision setting.

The finding that participation in the school-based group appeared to transform parent–school relationships from adversarial to collaborative is particularly significant given the extensive literature documenting how parents of children with emotional and behavioural difficulties frequently experience interactions with schools as stigmatising and blame-laden [3]. Several parents described this relational transformation explicitly. This shift can be understood through the lens of epistemic trust: when the school—previously experienced as a source of judgment—becomes a context in which parents feel mentalised and supported, the communicative channel between parent and school may be opened in ways that support ongoing collaboration around the child’s needs [35].

Crucially, this shift in the parent–school relationship may itself function as a therapeutic mechanism with direct implications for child wellbeing. When parents come to experience the school as a containing, trustworthy institution rather than a source of blame or judgement, this transformation in their relational stance may be communicated—explicitly and implicitly—to their child. A child whose parent conveys confidence in the school’s good intentions and safety is more likely to experience the school environment as emotionally containing, which in turn may support their capacity for affect regulation, their willingness to take interpersonal risks in learning, and their felt sense of safety within the educational context. In this sense, the parent–institution relationship is not merely a byproduct of successful intervention but a potentially active therapeutic mechanism through which the benefits of parental mentalization may be transmitted to the child’s subjective experience of safety and containment at school.

The present findings complement evidence that school-based mentalization interventions can improve student socio-emotional competencies [24] by demonstrating that mentalization-based approaches can be extended to parents within educational contexts. Together, these strands of evidence point toward the potential for whole-school mentalization approaches that address both student and parent capacities—integrated models that could create coherent, systemic supports for families and schools working in partnership.

### 6.7. Parent-Reported Child Outcomes

Although the study did not include independent measures of child functioning, parents reported observable improvements in their children—including increased calmness, fewer escalations and greater willingness to share feelings—consistent with evidence linking PRF to children’s emotional regulation and behavioural outcomes [38]. These reports are consistent with theoretical models of relational reciprocity in which improved parental mentalizing enhances the contingency and attunement of caregiving responses, which in turn scaffolds children’s developing regulatory capacities [36,40]. The most striking account—a child’s transformation from chronic school refusal to enthusiastic engagement—suggests that enhancements in parental mentalizing may have particularly powerful effects for children whose difficulties have led to sustained educational disengagement, though causal claims require future research incorporating direct, independent child outcome measures.

## 7. Limitations

Several limitations of the present study must be acknowledged. First, the small sample size (N = 12) substantially limits statistical power and the generalisability of findings. While the observed effect sizes were large and exceeded those typically reported in similar group-based parental interventions, small samples are susceptible to sampling error, and caution is warranted in interpreting their magnitude. The absence of a control or comparison group means that it is not possible to attribute observed changes to the intervention itself; alternative explanations, including maturation, regression to the mean, and demand characteristics, cannot be excluded. Furthermore, the single-school design—with all participants drawn from one alternative provision setting in London—limits generalisability to other educational contexts, cultural populations, or socio-economic groups. The school operates within a distinctive whole-school mentalization framework and benefits from highly experienced practitioners; these conditions may not be readily replicable in typical school settings without substantial investment in practitioner training and school culture development. Findings should therefore be treated as context-specific and hypothesis-generating, rather than as evidence of generalisable effects.

Second, the absence of a control or comparison condition precludes causal inference; future research should utilise randomised controlled designs to isolate intervention effects.

Third, the retrospective pretest–posttest design represents a pragmatic choice necessitated by the constraints of conducting research within an active school-based intervention, but it introduces known methodological vulnerabilities. Retrospective pretest designs are susceptible to recall bias and may overstate change when participants are aware of desired outcomes or when the intervention itself has influenced how participants reconstruct their prior states [25]. Although participants reported high confidence in their retrospective responses, this confidence does not eliminate the possibility that the intervention experience itself shaped how parents remembered their pre-intervention functioning. The qualitative data are particularly valuable in this context, as parents’ detailed, contextualised narratives of specific behavioural changes—rather than generalised self-assessments—provide a complementary evidence base that is less susceptible to the biases inherent in retrospective self-report.

Fourth, reliance on self-report measures limits the strength of conclusions that can be drawn about behavioural and relational change; incorporation of observational assessments of parent–child interaction and multi-informant data would strengthen claims regarding behavioural outcomes.

Fifth, while the PRFQ is a widely used and psychometrically adequate measure of parental reflective functioning, it is a brief self-report instrument comprising 18 items, and as such, may not capture the depth and complexity of reflective functioning as assessed by interview-based measures such as the Parent Development Interview (PDI) [36] or observational coding systems. Self-report measures of mentalizing are vulnerable to social desirability bias and may assess participants’ knowledge of mentalizing concepts rather than their capacity to mentalize in real-time, affectively charged interactions.

Sixth, the absence of extended follow-up assessment means that the durability of intervention effects remains unknown; understanding whether gains are maintained over time and what supports maintenance is crucial for clinical and educational application. Seventh, the study did not include direct, independent measures of child functioning. While parents reported perceived changes in their children, independent assessment of child outcomes would provide stronger evidence for the downstream effects of parental change. Finally, in the absence of measures assessing group processes, it is not possible to disentangle the relative contribution of group-level therapeutic dynamics from the psychoeducational and mentalization-based content of the intervention, although participants’ experiences suggest that the group context and facilitation played a central role in perceived change.

### 7.1. Implications for Practice

Despite these limitations, the findings offer several important implications for practice. First, they support the feasibility of delivering mentalization-based parent support within school settings, extending the reach of reflective and psychoeducational interventions beyond clinical service contexts, where parental access is often limited. School-based delivery can provide an accessible platform for engaging families who may not otherwise access clinical services and may help reframe school–parent relationships from adversarial to collaborative. The apparent synergy between psychoeducational content and mentalization-based practice suggests that informational material should be used to facilitate the development of reflective capacity rather than serve as a standalone strategy. Moreover, the qualitative data highlight the significance of group process—including relational safety, peer support and facilitator mentalizing—indicating that attention to group climate and facilitator skill is as important as content delivery. These insights also point to the value of maintaining consistency in session structure and scheduling, which supports parent engagement and the gradual development of reflective skills. Furthermore, tailoring discussions to parents’ lived experiences, while allowing space for peer exchange, appears to enhance learning and confidence. Overall, the findings suggest that successful school-based interventions balance structured psychoeducational content with opportunities for reflection, dialogue, and mutual support, creating an environment that fosters both parental growth and improved relationships with their children and the school.

### 7.2. Implications for Future Research

The present findings generate several specific directions for future research that would build systematically on this preliminary work. First, and most critically, randomised controlled trials with active comparison conditions are needed to establish causal relationships. Given the ethical and practical constraints of school-based research—where withholding a promising intervention from families may be unacceptable—stepped-wedge cluster randomised designs represent a particularly appropriate methodology, as they allow all participants to eventually receive the intervention while maintaining the capacity for causal inference [44].

Second, pragmatic or naturalistic trials embedded within routine school practice would address the question of effectiveness under real-world conditions, complementing the efficacy-focused designs typically employed in clinical research. Such trials should incorporate process evaluation alongside outcome assessment, examining how implementation factors—including facilitator training requirements, adaptations for diverse educational settings, parent engagement patterns and systemic school-level supports—moderate intervention effects.

Third, research examining mediators and moderators of intervention effects would advance understanding of for whom and through what mechanisms the intervention is most effective. This should include measures of group environment quality (e.g., group cohesion, therapeutic alliance), facilitator adherence to mentalizing principles, and parent characteristics (e.g., baseline reflective functioning, attachment history, neurodevelopmental status) that may moderate treatment response.

Fourth, longitudinal designs with extended follow-up at 6- and 12-month intervals are essential to assess the durability of intervention effects and to identify factors that support maintenance or attenuation of gains over time.

Fifth, incorporating interview-based or observational measures of parental reflective functioning alongside the PRFQ, and direct child outcome assessments including teacher-reported behaviour, observational measures of parent–child interaction, and child self-report where appropriate, would strengthen causal claims about mechanisms linking parental change to child outcomes.

Finally, investigation of whole-school mentalization approaches that integrate student-focused mentalization-based interventions [24] with parent groups represents a promising direction, as such integrated models could create coherent, systemic supports that address both child and family capacities within a unified theoretical framework.

## 8. Conclusions

The present study provides preliminary evidence that an integrated parent learning group combining psychoeducation, mentalization-based practice and peer support is feasible and associated with meaningful improvements in parental reflective functioning and self-efficacy among parents of children with neurodevelopmental and conduct difficulties in an alternative provision school setting. While the large effect sizes are encouraging, they should be interpreted cautiously given the small sample, retrospective design and absence of a control group. The qualitative findings provide critical complementary evidence, illuminating the process mechanisms—epistemic trust, facilitator modelling, peer-mediated reflective processes and relational reciprocity—through which change appears to have occurred.

These findings contribute to an evolving but still limited evidence base for group-delivered mentalization interventions, extending this literature into the underexplored context of alternative provision schools. They align with prior research on the modifiability of parental reflective functioning and complement evidence for school-based mentalization interventions, while highlighting the importance of relational and group-process factors that are often underemphasised in intervention research focused primarily on content delivery.

By situating parent support within the school community, such interventions may simultaneously enhance parenting capacity and strengthen home–school partnerships—dual goals of particular importance for families whose children present with complex emotional and behavioural needs. Replication with more rigorous designs—including stepped-wedge trials, longer-term follow-up, observational measures and independent child outcome assessment—is essential to establish the robustness and generalisability of these preliminary findings. Nevertheless, the present study offers a promising model for supporting parents and families within educational contexts and generates clear, testable hypotheses about the mechanisms through which integrated mentalization-informed interventions may operate.

## Data Availability

Data are available from the corresponding author upon reasonable request. The data are not publicly available due to privacy or ethical restrictions.
